# Effect of salidroside on lung injury by upregulating peroxisome proliferator-activated receptor γ expression in septic rats

**DOI:** 10.3892/etm.2014.1629

**Published:** 2014-03-21

**Authors:** MING-WEI LIU, MEI-XIAN SU, LAN-FANG QIN, XU LIU, MAO-LI TIAN, WEI ZHANG, YUN-HUI WANG

**Affiliations:** 1 Department of Emergency, The First Hospital Affiliated To Kunming Medical University, Kunming, Yunnan 650032, P.R. China; 2 Surgical Intensive Care Unit, The Second Hospital Affiliated To Kunming Medical University, Kunming, Yunnan 650106, P.R. China; 3 Department of Infectious Diseases, Yan’an Hospital Affiliated To Kunming Medical University, Kunming, Yunnan 650051, P.R. China

**Keywords:** sepsis, peroxisome proliferator-activated receptor γ, salidroside, rat, cecal ligation and puncture, lung injury

## Abstract

Successful drug treatment for sepsis-related acute lung injury (ALI) remains a major clinical problem. Thus, the aim of the present study was to investigate the beneficial effects of salidroside on ameliorating cecal ligation and puncture (CLP)-induced lung inflammation. Rats underwent CLP surgery to induce ALI and 800 mg/kg salidroside (i.v.) was administered 24 h after the CLP challenge. Subsequently, biochemical changes in the blood and lung tissues, as well as morphological and histological alterations in the lungs, that were associated with inflammation and injury were analysed. CLP was shown to significantly increase the serum levels of plasma tumour necrosis factor-α and interleukin-6, -1β and-10. In addition, CLP increased pulmonary oedema, thickened the alveolar septa and caused inflammation in the lung cells. These changes were ameliorated by the administration of 800 mg/kg salidroside (i.v.) 24 h after the CLP challenge. This post-treatment drug administration also significantly attenuated the lipopolysaccharide-induced activation of nuclear factor-κβ and increased the release of peroxisome proliferator-activated receptor γ in the lung tissue. Therefore, salidroside administered following the induction of ALI by CLP significantly prevented and reversed lung tissue injuries. The positive post-treatment effects of salidroside administration indicated that salidroside may be a potential candidate for the management of lung inflammation in CLP-induced endotoxemia and septic shock.

## Introduction

Acute respiratory distress syndrome (ARDS), a clinically important complication of severe acute lung injuries (ALIs) in humans, is a significant cause of morbidity and mortality in critically ill patients (1). Infectious etiologies, including sepsis and pneumonia, are the most common causes of ALI (1,2). Histologically, ALI in humans is characterised by a severe acute inflammatory response in the lungs and neutrophilic alveolitis (1,3). The physiological hallmark of ARDS is the disruption of the alveolar-capillary membrane barrier, leading to the development of noncardiogenic pulmonary oedema, in which a proteinaceous exudate floods the alveolar spaces, impairs gas exchange and precipitates respiratory failure (1,4,5). Despite numerous studies investigating the early diagnostic and pathogenetic factors of ALI, the current management of ALI is predominantly supportive since specific therapies have not yet been identified (6,7). Thus, novel strategies are required for achieving effective treatment of ALI in clinical practice.

*Rhodiola rosea* (*R. rosea*) has been widely used in traditional Chinese medicine for centuries (8). A previous study confirmed that the plant exhibits various pharmacological effects, including improving exercise endurance and fatigue, preventing high altitude sickness, promoting blood circulation, eliminating toxins from the body and treating various endemic diseases (9). *R. rosea* also has been demonstrated to have antimicrobial and anticancer effects (9). Salidroside (p-hydroxyphenethyl-β-D-glucoside; C_14_H_20_O_7_) is a phenylpropanoid glycoside that can be extracted from *R. rosea* and is regarded as the most important bioactive component in the species (10). Salidroside has been reported to have various pharmacological effects, including anti-aging, anticancer, hepatoprotective, antiviral and antioxidative. In addition, salidroside has been shown to suppress the release of prostaglandin E2 *in vitro* (11,12). A previous study identified that *R. rosea* suppresses the inflammatory response (13); however, the specific mechanism remains unknown.

Peroxisome proliferator-activated receptors (PPARs) are transcription factors belonging to the nuclear receptor superfamily. The three known PPAR subtypes, α, γ and δ, have various tissue distributions and are associated with selective ligands. PPAR-α is predominantly expressed in tissues that demonstrate high catabolism for fatty acids, including the liver, heart, kidney and muscle tissues. PPAR-γ is expressed to the greatest extent in white adipose tissue, where it plays a major regulatory role in adipocyte differentiation and lipid metabolism. PPAR-δ is ubiquitously expressed, thus, to date, no specific role has been demonstrated for this isoform. Contradictory results have been generated concerning the role of PPAR-γ in inflammatory models *in vivo*. The activation of PPAR-γ has been shown to inhibit inflammation in a model of inflammatory bowel disease (14) and a mouse model of atherosclerosis (15).

Therefore, in the present study, the potential role of salidroside for the treatment of ALI was evaluated using a rat model of CLP-induced ALI.

## Materials and methods

### Rats

Adult male Sprague-Dawley rats, weighing 250±25 g (supplied by The Field Surgery Institute, Kunming Medical University, Kunming, China), were housed in 12-h light-dark conditions with free access to water and standard laboratory chow. The animal procedures were performed in strict accordance with National Institutes of Health guidelines, which were approved by the Ethics Committee of Kunming Medical University.

### Reagents

A TRIzol kit was obtained from Gibco-BRL (Carlsbad, CA, USA) and a reverse transcription kit was purchased from Takara Biotechnology Co. Ltd. (Dalian, China). A polymerase chain reaction (PCR) amplification reagent kit and the DNA ladder marker were obtained from Sangon Biological Engineering Co. Ltd. (Shanghai, China). β-actin was obtained from Santa Cruz Biotechnology, Inc. (Santa Cruz, CA, USA) and tumour necrosis factor-α (TNF-α), interleukin (IL)-6, -1β and -10 enzyme-linked immunosorbent assay (ELISA) kits were obtained from Pierce Biotechnology Inc. (Rockford, IL, USA). Salidroside (99% purity) was acquired from the National Institute for the Control of Pharmaceutical and Biological Products (Beijing, China).

### Model and groups

Using a random number table, 80 rats were randomly divided into four groups: Normal control, sham-operation (sham), sepsis model (model) and salidroside treatment (treatment) groups (n=20 per group). The sepsis model was induced by cecal ligation and puncture (CLP). Briefly, the animals were deprived of food, but water was permitted for 6 h prior to surgery. Under light ether anaesthesia, a laparotomy was performed through a midline abdominal incision. The cecum was punctured twice at different sites with an 18-gauge needle and gently compressed until faeces were extruded. The bowel was then returned to the abdomen and the abdominal incision was closed in two layers. Animals in the model and treatment groups were treated with 5 ml normal saline per 100 g body weight subcutaneously at the completion of surgery to replace the extracellular fluid sequestered during peritonitis (16). Animals in the sham group underwent sham surgery, in which the cecum was neither ligated nor punctured. Starting at 8 h prior to surgery, animals in the treatment group were injected with 800 mg/kg salidroside every 8 h, while the animals in the normal control, sham and model groups were administered the same volume of normal saline. Subsequently, the animals in each group were anaesthetised with ether 24 h post-surgery, and the right internal carotid artery was isolated. Blood was extracted (5 ml), centrifuged to collect the supernatant, dispensed into two sterile tubes, sealed with sealing glue and placed in freezer at −20°C for examination. All the animals were sacrificed 24 h following surgery via anesthesia and tissue samples were collected for further tests.

### Extraction of RNA

For the isolation of lung tissue RNA, the rats were humanely sacrificed and under aseptic conditions, the lung tissue was removed and immediately frozen in liquid nitrogen. Prior to RNA extraction, the lung samples were homogenised in TRIzol reagent (Invitrogen, Carlsbad, CA, USA) using a Mixer Mill 301. The total RNA was extracted according to the manufacturer’s instructions. The RNA samples were electrophoresed in agarose gel and visualised with ethidium bromide for quality control.

### cDNA synthesis and quantitative PCR (qPCR)

RNA (3 μg) underwent reverse transcription with reverse transcriptase for 1 h at 37°C to synthesise cDNA. Quantitative changes in the mRNA expression levels were assessed with qPCR (CFX; Bio-Rad, Hercules, CA, USA) using SYBR Green detection, consisting of the SYBR Green PCR Master mix (Aria-tous, Iran). The PCR master mix consisted of 0.5 units *Taq* polymerase, 2-μl samples of each primer and 3-μl samples of each cDNA sample in a final volume of 20 μl. The amplifications were repeated three times. The oligonucleotide primer sequences are presented in Table I. β-actin was used as an endogenous control and each sample was normalised on the basis of its β-actin content. The relative quantification of the mRNA expression levels of the target genes was calculated using the 2^−ΔΔCt^ method (17).

### Western blot analysis

Homogenised lung tissue was preserved in a protease-inhibitor solution (Complete Mini; Roche). Western blot analyses were performed for PPAR-γ, inhibitor-κβ (Iκβ) and NF-κβ p65 using 20 μg protein. The PPAR-γ specimens were separated by electrophoresis in a 10% Bis-Tris gel, while the NF-κβ p65 and Iκβ specimens were separated by electrophoresis in a 3–8% Tris-acetate gel. Following electrophoresis, the proteins were transferred to a nitrocellulose membrane. Primary antibodies against β-actin, PPAR-γ, phospho-PPAR-γ (Cell Signaling Technology, Inc., Danvers, MA, USA), NF-κβ p65 and Iκβ (R&D Systems, Minneapolis, MN, USA) were applied overnight at 4°C in Tris-buffered saline, which was followed by the addition of anti-rabbit horseradish peroxidase-conjugated secondary antibodies (Millipore, Billerica, MA, USA) for 1 h at room temperature (RT). Chemiluminescence detection was performed with the Western Lightning detection reagent (Perkin-Elmer, Waltham, MA, USA).

### Immunohistochemistry

Immunostaining was performed on the lung sections following antigen retrieval using Retrievagen A (Zymed, South San Francisco, CA, USA) at 100°C for 20 min and the quenching of endogenous peroxidases with 3% H_2_O_2_. The sections were blocked with 2% bovine serum albumin in phosphate-buffered saline, which was followed by staining with primary anti-PPAR-γ, anti-NF-κβ p65 and Iκβ (BD Pharmingen, San Jose, CA, USA) antibodies at RT for 1 h. The sections were washed and secondary antibodies (R&D Systems) were applied, after which the tissues were developed using Vectastain ABC (Vector Laboratories, Burlingame, CA, USA) and 3,3′-diaminobenzidine (Vector Laboratories). Using Image-Pro Plus image analysis software (Media Cybernetics, Inc., Rockville, BD, USA), the PPAR-γ, Iκβ and NF-κβ p65 positive expression levels in the lung tissue were calculated and expressed in positive units.

### Cytokine content measurement in the serum

Serum levels of TNF-α, IL-1β, IL-6 and IL-10 in the rats were measured using ELISA kits, according to the manufacturer’s instructions (R&D Systems).

### Determination of the total number of cells and neutrophils

According to a previously described procedure (18), the analysis of bronchoalveolar lavage fluid (BALF) was performed by instilling 0.9% NaCl containing 0.6 mmol/l ethylenediaminetetraacetic acid in two separate 0.5 ml aliquots. The fluid was recovered by gentle suction and placed on ice for immediate processing. An aliquot of the BALF was processed immediately for total and differential cell counts. The remainder of the BALF was centrifuged and the supernatant was removed aseptically and stored in individual aliquots at −70°C. The total cell counts in the BALF were determined using a haemocytometer and the number of neutrophils was calculated as the percentage of neutrophils multiplied by the total number of cells in the BALF sample. All analyses were performed in a blind manner.

### Elastase activity

Elastase activity was measured using an EnzChek Elastase Assay kit (E-12056; Molecular Probes Europe, Leiden, The Netherlands). Absorbance was measured at 515 nm with a microplate reader (Infinite 200; Tecan, Männedorf, Switzerland).

### Albumin concentration in the BALF

Albumin content in the BALF supernatants was assessed using an ELISA kit for albumin (E91028Mu; Uscn Life Science, Inc., Wuhan, China). Absorbance was measured at 450/540 nm using a microplate reader.

### Measurement of the pulmonary oedema

The right lungs of the rats were removed and the wet weights were obtained. The lungs were weighed again following drying for three days at 55°C. The wet to dry (W/D) ratio was calculated as follows: W/D ratio = (wet weight - dry weight)/dry weight (19). The lung water content was calculated as the wet weight minus the dry weight and the wet weight ratio of the lung tissue multiplied by 100.

### Histopathological examination

Lung tissue was fixed in 10% formalin for 24 h, which was followed by dehydration. The lung tissue was embedded in paraffin wax, sectioned into 5-μm-thick slices and stained with haematoxylin and eosin. Microphotography of the lung sections was captured with a light microscope (Olympus, Tokyo, Japan). The severity of the ALI was scored in a blind manner, as previously described (6), according to the categorical degree scoring (zero, minimal or no damage; four, severe damage) of alveolar congestion, haemorrhaging, cell infiltration into the airspace or vessel wall and thickness of the alveolar wall. The mean score of five random areas per section per animal was used for data analysis.

### Statistical analysis

Quantitative data are presented as the mean ± standard error of the mean (SEM) of at least three independent experiments. The histological injury scoring data were analysed by analysis of variance (ANOVA) followed by the Kruskal-Wallis nonparametric test for comparison, which was then presented as a box-and-whisker plot. The remaining data were analysed by ANOVA and then with the Newman-Keuls test for comparison. For comparisons among the groups, the unpaired Student’s t-test was used (GraphPad Prism, GraphPad Software Inc., San Diego, CA, USA), in which P<0.05 was considered to indicate a statistically significant difference.

## Results

### Salidroside upregulates the expression levels of PPAR-γ and Iκβ and blocks NF-κβ p65 expression in lung tissue

**Figure 1:**
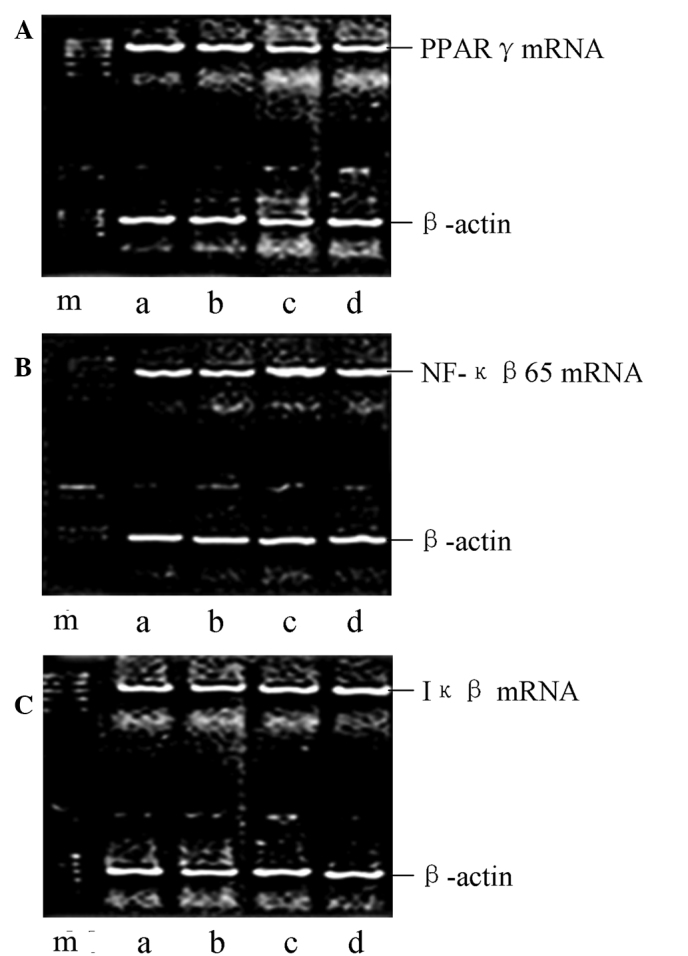
Effect of salidroside on the mRNA expression levels of PPAR-γ, Iκβ and NF-κβ p65 in the lung tissue of CLP-ALI rats, as determined by qPCR. Representative gels show the mRNA expression levels of (A) PPAR-γ, (B) NF-κβ p65 and (C) Iκβ in the four groups of rats: m, marker; a, normal control; b, sham surgery; c, model; and d, treatment groups. PPAR-γ, peroxisome proliferator-activated receptor γ; NF-κβ, nuclear factor-κβ; CLP, cecal ligation and puncture; ALI, acute lung injury; qPCR, quantitative polymerase chain reaction; Iκβ, inhibitor-Iκβ.

**Figure 2:**
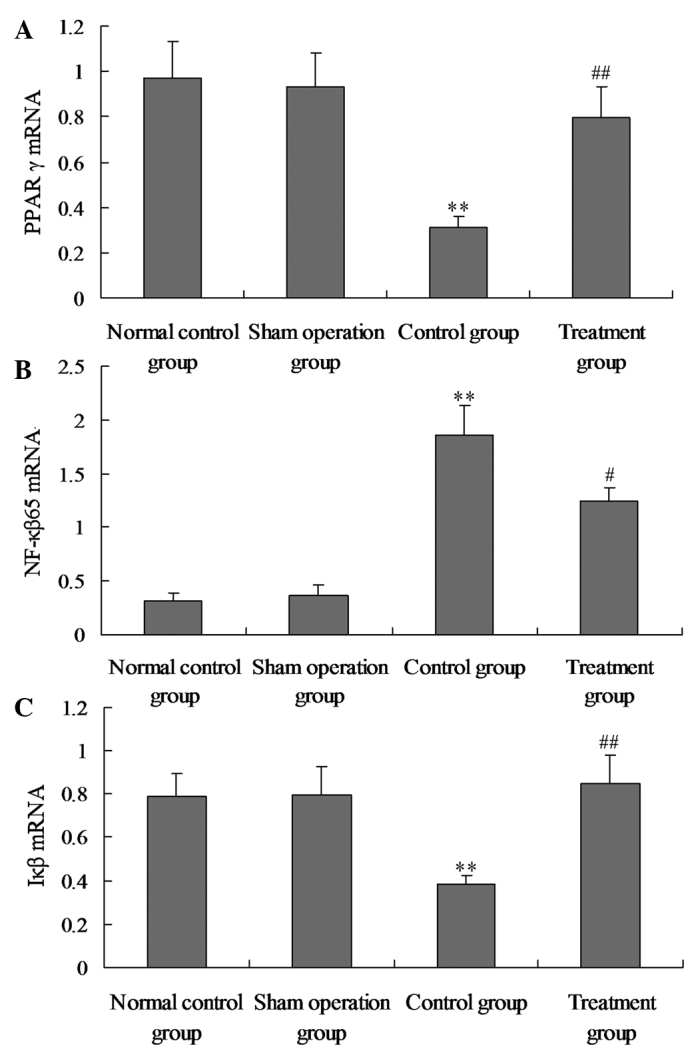
Administration of salidroside increased the mRNA expression levels of PPAR-γ and Iκβ and inhibited the mRNA expression of NF-κβ p65 in the lung tissue of CLP-ALI rats, as determined by qPCR. A statistical summary of the densitometric analysis of (A) PPAR-γ, (B) NF-κβ p65 and (C) Iκβ mRNA expression levels in the four groups of rats. Data are presented as the mean ± standard deviation of one experiment consisting of three replicates. The experiments were performed in triplicate. ^*^P<0.05 and ^**^P<0.01, vs. normal control and sham surgery groups; ^#^P<0.05 and ^##^P<0.01, vs. control group. PPAR-γ, peroxisome proliferator-activated receptor γ; NF-κβ, nuclear factor-κβ; CLP, cecal ligation and puncture; ALI, acute lung injury; qPCR, quantitative polymerase chain reaction; Iκβ, inhibitor-Iκβ.

**Figure 3:**
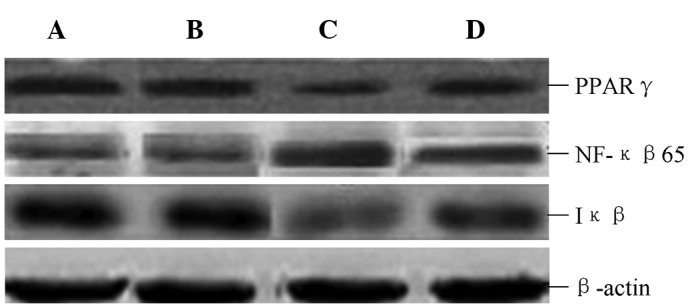
Administration of salidroside enhanced the protein expression levels of PPAR-γ and Iκβ and inhibited the protein expression of NF-κβ p65 in the lung tissue of CLP-ALI rats. The groups of rats were challenged with CLP and treated with salidroside 24 h later. PPAR-γ, NF-κβ p65 and Iκβ levels were assayed by western blotting and representative western blots show the protein expression levels of PPAR-γ, NF-κβ p65 and Iκβ in the four groups of rats: (A) Normal control; (B) sham surgery; (C) model and (D) treatment groups. PPAR-γ, peroxisome proliferator-activated receptor γ; NF-κβ, nuclear factor-κβ; CLP, cecal ligation and puncture; ALI, acute lung injury; Iκβ, inhibitor-Iκβ.

**Figure 4:**
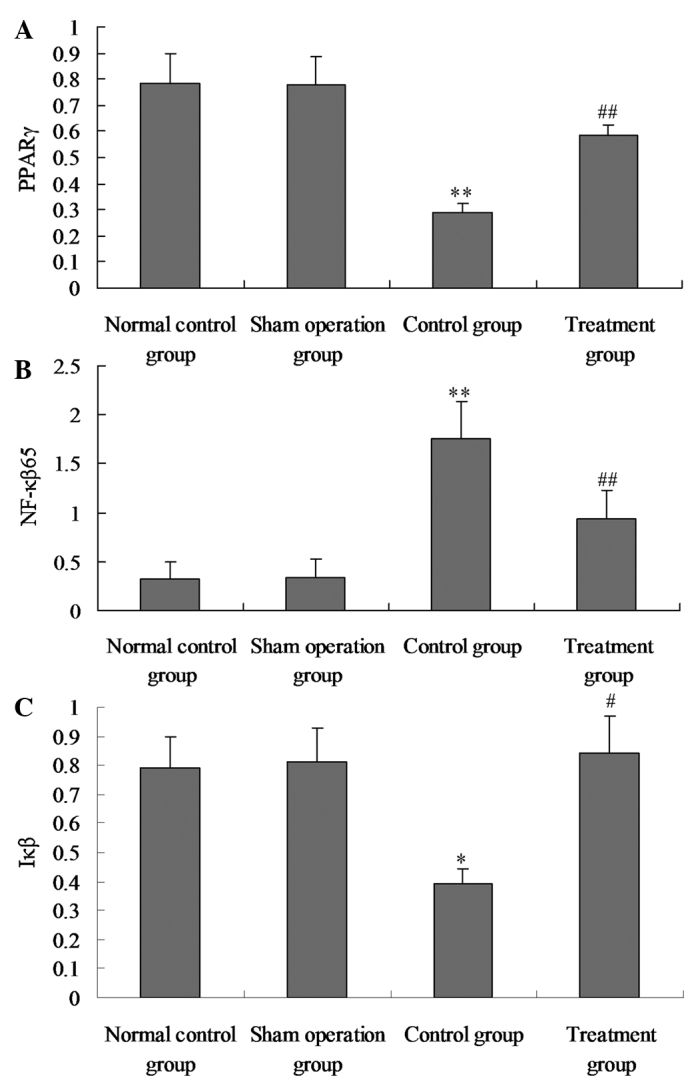
Administration of salidroside enhanced the protein expression levels of PPAR-γ and Iκβ and inhibited the protein expression of NF-κβ p65 in the lung tissue of CLP-ALI rats. The groups of rats were challenged with CLP and treated with salidroside 24 h later. PPAR-γ, NF-κβ65 and Iκβ were assayed by western blotting and a statistical summary of the densitometric analysis of (A) PPAR-γ, (B) NF-κβ p65 and (C) Iκβ protein expression levels in the four groups of rats is shown. Data are presented as the mean ± standard deviation of one experiment consisting of three replicates. The experiments were performed in triplicate. ^**^P<0.01, vs. normal control and sham surgery groups. ^#^P<0.05 and ^##^P<0.01, vs. control group. PPAR-γ, peroxisome proliferator-activated receptor γ; NF-κβ, nuclear factor-κβ; CLP, cecal ligation and puncture; ALI, acute lung injury; Iκβ, inhibitor-Iκβ.

To assess the potential role of salidroside in CLP-induced ALI, the mRNA and protein expression levels of PPAR-γ, NF-κβ p65 and Iκβ in the lung tissue of the lipopolysaccharide (LPS)-induced ALI rats were determined using qPCR and western blot analysis at 24 h after the CLP challenge. As shown in Figs. 1–4, the expression levels of PPAR-γ and Iκβ were markedly reduced and the NF-κβ p65 expression levels were markedly enhanced in the model group compared with those in the normal control and sham groups. However, following the administration of salidroside, the expression levels of PPAR-γ and Iκβ were markedly upregulated and NF-κβ p65 expression levels were significantly decreased. In combination, these observations indicated that salidroside may be involved in the increased expression levels of PPAR-γ and Iκβ and the reduced NF-κβ p65 expression levels in CLP-induced ALI. A negative correlation was shown to exist between PPAR-γ and NF-κβ p65 mRNA expression levels, as well as between PPAR-γ and NF-κβ p65 protein (r=−0.452, P<0.05; r=0.613, P<0.05).

### Salidroside increases PPAR-γ and Iκβ activation in lung tissue, and inhibits NF-κβ p65 activation in lung tissue

**Figure 5:**
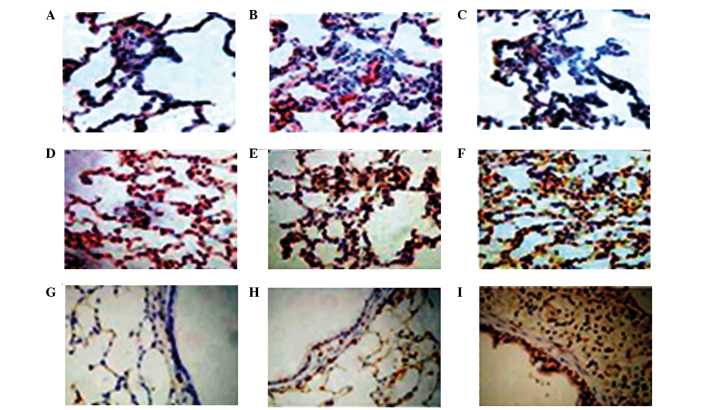
Effect of the administration of salidroside on the PPAR-γ, NF-κβ p65 and Iκβ positive expression levels in the lung tissue of CLP-ALI rats. The groups of rats were challenged with CLP and treated with salidroside 24 h later. Immunostaining was performed on the lung sections following antigen retrieval using Retrievagen A. Representative immunostaining images show the positive expression levels of PPAR-γ, NF-κβ and Iκβ in three groups of rats (immunofluorescence staining; magnification, ×200). Positive expression levels of (A–C) NF-κβ p65 (A, sham surgery group; B, model group; C, treatment group); (D–F) PPAR-γ (D, sham surgery group; E, model group; F, treatment group); (G–I) Iκβ (G, sham surgery group; H, model group; I, treatment group). PPAR-γ, peroxisome proliferator-activated receptor γ; NF-κβ, nuclear factor-κβ; CLP, cecal ligation and puncture; ALI, acute lung injury; Iκβ, inhibitor-Iκβ.

**Figure 6:**
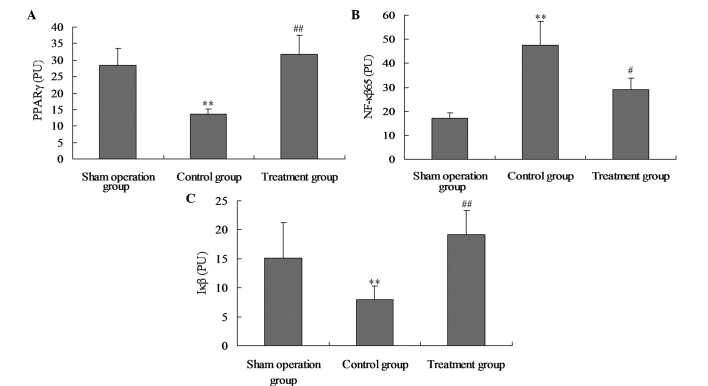
Effect of salidroside on (A) PPAR-γ, (B) NF-κβ p65 and (C) Iκβ positive expression levels in the lung tissue of CLP-ALI rats. The groups of rats were challenged with CLP and treated with salidroside 24 h later. Immunostaining was performed on the lung sections following antigen retrieval using Retrievagen A. Using Image-Pro Plus image analysis software, the PPAR-γ, Iκβ and NF-κβ p65 positive expression levels in lung tissue were calculated. Data are presented as the mean ± standard deviation of one experiment consisting of three replicates. The experiments were performed in triplicate. ^*^P<0.05 and ^**^P<0.01, vs. normal control and sham surgery groups. ^#^P<0.05 and ^##^P<0.01, vs. control group. PPAR-γ, peroxisome proliferator-activated receptor γ; NF-κβ, nuclear factor-κβ; CLP, cecal ligation and puncture; ALI, acute lung injury; Iκβ, inhibitor-Iκβ.

To study the effect of salidroside on the positive expression of PPAR-γ, NF-κβ p65 and Iκβ in CLP-induced ALI, immunohistochemical staining was performed on the lung sections. As shown in Figs. 5 and 6, in the model group, the activation of PPAR-γ and Iκβ was significantly suppressed and the activation of NF-κβ p65 was significantly increased at 24 h after the CLP challenge, as compared with the sham group. However, in the CLP-induced rats that received salidroside, the positive expression levels of PPAR-γ and Iκβ were significantly increased and the NF-κβ p65 positive expression levels were markedly inhibited compared with the model group. Therefore, salidroside may suppress the positive expression of NF-κβ p65 and promote the positive expression of PPAR-γ and Iκβ.

### Salidroside reduces the production of proinflammatory cytokines

**Figure 7:**
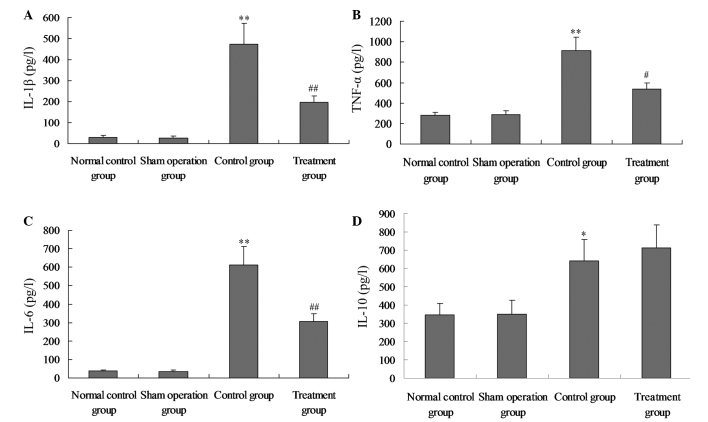
Administration of salidroside attenuated the CLP-induced inflammatory response. Groups of rats were challenged with CLP and treated with salidroside 24 h later. Plasma levels of (A) TNF-α, (B) IL-6, (C) IL-1β and (D) IL-10 were determined by ELISA. Data are presented as the mean ± standard deviation of one experiment consisting of three replicates. The experiments were performed in triplicate. ^*^P<0.05 and ^**^P<0.01, vs. normal control and sham surgery groups. ^#^P<0.05 and ^##^P<0.01, vs. control group. TNF-α, tumour necrosis factor-α; IL, interleukin; CLP, cecal ligation and puncture; ELISA, enzyme-linked immunosorbent assay.

To further assess the anti-inflammatory effect of salidroside, the levels of proinflammatory and anti-inflammatory cytokines in the plasma were detected. The level of proinflammatory cytokines, including TNF-α, IL-1β and IL-6, were all significantly elevated in the plasma in response to the LPS challenge (Fig. 7A–C; P<0.05). By contrast, the administration of salidroside effectively reduced the levels of proinflammatory cytokines (P<0.05) as shown in Fig. 7A–C. Consistent with these observations, the administration of salidroside after a 24 h interval further increased the level of anti-inflammatory cytokines to relatively high levels in the plasma (Fig. 7D).

### Salidroside attenuates the acute CLP-induced pulmonary inflammation

**Figure 8:**
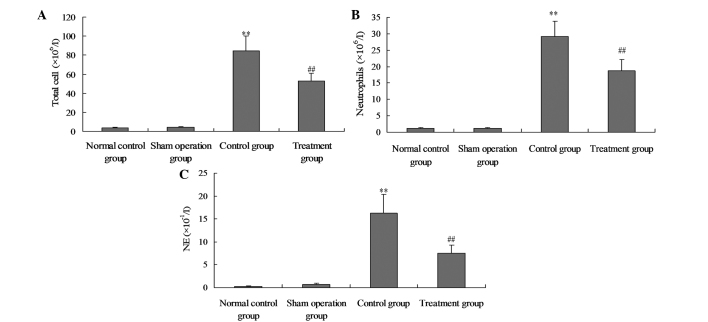
Administration of salidroside attenuated CLP-induced pulmonary inflammation. Groups of rats were challenged with LPS and treated with salidroside 24 h later. Salidroside was administered three times at 8 h intervals. (A) Total cell, (B) neutrophil and (C) NE counts in the BALF were detected to evaluate the lung airspace inflammation at 24 h following the LPS challenge. Data are presented as the mean ± standard deviation of one experiment consisting of three replicates. Experiments were performed in triplicate. ^**^P<0.01, vs. normal control and sham surgery groups; ^##^P<0.01, vs. control group. NE, neutrophil elastase; LPS, lipopolysaccharide; CLP, cecal ligation and puncture; BALF, bronchoalveolar lavage fluid.

To investigate the possible mechanism underlying the protective effect of salidroside on CLP-induced ALI, the total cell counts, neutrophil counts and neutrophil elastase levels were analysed in the BALF of the rats treated with CLP with or without salidroside treatment. As shown in Fig. 8, the total numbers of inflammatory cells, neutrophils and NE in the BALF were markedly increased following the administration of CLP. Following the administration of salidroside, the total numbers of inflammatory cells, neutrophils and NE in the BALF were significantly reduced.

### Salidroside reduces CLP-induced lung permeability

**Figure 9:**
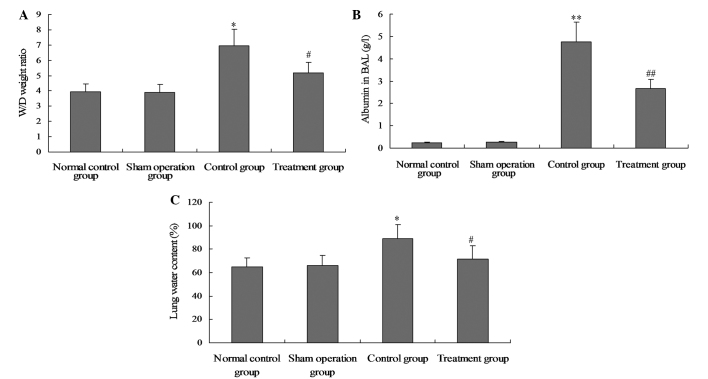
Administration of salidroside attenuated CLP-induced lung permeability. Groups of rats were challenged with LPS and treated with salidroside 24 h later. (A) W/D lung weight ratio, (B) albumin concentration and (C) water content were measured in the lung tissue. Data are presented as the mean ± standard deviation of one experiment consisting of three replicates. The experiments were performed in triplicate. ^**^P<0.01, vs. normal control and sham surgery groups; ^##^P<0.01, vs. control group. W/D, wet to dry; BALF, bronchoalveolar lavage fluid; CLP, cecal ligation and puncture.

Total protein concentration in the BALF, the W/D lung weight ratio and the water content in the lung tissue were determined to evaluate the integrity of the alveolar-capillary membrane barrier and assess the pulmonary vascular leakage as a marker of ALI. As shown in Fig. 9, BALF albumin concentration, W/D lung weight ratio and the water content in the lung tissue of the model group were markedly increased compared with the normal control and sham groups. However, following salidroside treatment, the albumin concentration in the BALF, the W/D lung weight ratio and the water content decreased significantly.

### Salidroside ameliorates the histopathological changes in the lungs of CLP-ALI rats

**Figure 10:**
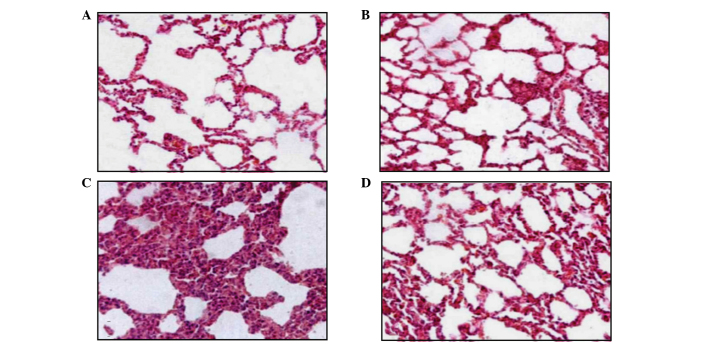
Administration of salidroside ameliorated the histopathological changes of the lungs in CLP-ALI rats. Histological evaluation of the therapeutic potential of salidroside on CLP-induced lung injury in rats was analysed at 24 h after the CLP challenge (haematoxylin and eosin staining; magnification, ×200). Representative images of the haematoxylin and eosin-stained lung sections from the four experimental groups are shown: (A) Normal control; (B) sham surgery; (C) model and (D) treatment groups. CLP, cecal ligation and puncture; ALI, acute lung injury; CLP, cecal ligation and puncture.

**Figure 11:**
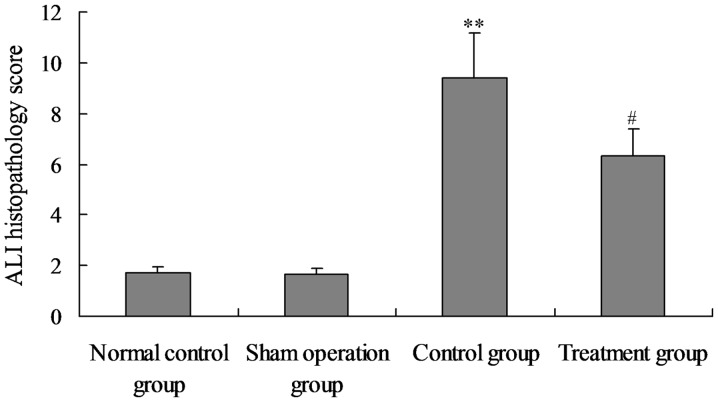
Administration of salidroside decreased the ALI histopathology score of the lungs in CLP-ALI rats. Histological evaluation of the therapeutic potential of salidroside on the LPS-induced lung injury in rats was analysed at 24 h after the LPS challenge and the lung injury scores were determined. ALI pathology scores are expressed as the mean ± standard deviation. ^**^P<0.01, vs. normal control and sham surgery groups. ^#^P<0.05, vs. control group. ALI, acute lung injury; CLP, cecal ligation and puncture; LPS, lipopolysaccharide.

To determine the effect of salidroside on histological lung injury, histopathological analysis was performed on the lung sections stained with haematoxylin and eosin. Histological analyses of the lungs following CLP exposure revealed a damaged alveolar structure with evident concretions and liquid draining within the bleeding inflammatory cells. In addition, the perivascular gap was widened and there were numerous alveolar stoma that were infiltrated by mononuclear inflammatory cells, including macrophages, plasma cells and neutrophils. The ALI pathology score also increased significantly compared with that in the normal control and sham groups. The lungs of the rats in the treatment group exhibited less severe damage without significant bleeding and the ALI pathology score was significantly reduced compared the model group (Figs. 10 and 11).

## Discussion

CLP-induced sepsis with acute suppurative peritonitis has been demonstrated to be a typical sepsis model with Gram-negative bacteria being the predominant source of infection (20). Gram-negative bacteria release numerous endotoxins and severe endotoxemia may activate the inflammatory cells and cause inflammatory reactions that lead to tissue and organ injury, dysfunction, and possibly mortality. Lung tissue is one of the most vulnerable tissues to endotoxemia. LPS causes ALI, which further develops into ARDS (21,22). In the present study, an endotoxemia rat model was created via the use of CLP to simulate sepsis-related lung injury in order to observe the effect of salidroside on ALI. The experimental results revealed that the rats exhibited varying degrees of lung tissue hyperaemia, haemorrhage, alveolar septal thickening, infiltration of the inflammatory cells and neutrophil accumulation, which are all pathological changes associated with ALI. These observations indicated that the model was successful.

ALI is an uncontrollable pulmonary inflammation caused by large amounts of inflammatory cells and cytokines. Under the effects of CLP, lung macrophages and neutrophils produce proinflammatory cytokines, including TNF-α and IL-1β, triggering the inflammatory reaction cascade (23). In the present study, the plasma levels of TNF-α, IL-1β, and IL-6 significantly increased 24 h after CLP surgery. When 800 mg/kg salidroside (i.v.) was administered 24 h after the CLP-induced injury, the plasma levels of the proinflammatory cytokines and lung inflammation decreased significantly. *In vitro* experiments demonstrated that 800 mg/kg salidroside (i.v.) reduced the levels of TNF-α, IL-1β and IL-6 secretion by lung macrophages. IL-10 is one of the most important anti-inflammatory cytokines and salidroside administration markedly increased the IL-10 concentration in the CLP-induced ALI rats. The administration of salidroside clearly inhibited the production of the proinflammatory cytokines, TNF-α, IL-1β and IL-6, and increased the IL-10 levels. Thus, salidroside improves the homeostasis of the cytokine network and the balance between the inflammatory and anti-inflammatory reactions associated with ALI.

PPARs are members of the nuclear receptor superfamily with three isomers existing in mammals: α, β and γ (24). Steroid, thyroid and retinoid hormones are ligands for the receptors. PPAR-γ is highly expressed in adipose tissue and its activation plays a key role in increasing systemic insulin sensitivity. PPAR-γ agonists are clinically used in the treatment of type 2 diabetes mellitus and metabolic syndrome. PPAR-γ has been shown to be constitutively expressed in numerous types of tissue, including lung tissue, where it has been hypothesised to play a protective role (25). In addition, PPAR-γ expression in macrophages and lymphocytes suppresses inflammatory responses, and PPAR-γ agonists inhibit the production of proinflammatory cytokines and regulate the process of inflammation by activating this nuclear receptor (20). PPAR-γ is a ligand-activated transcription factor, whose activation plays a role in controlling the inflammatory response. Several studies have demonstrated that the activation of PPAR-γ by specific ligands significantly improves survival rates in clinically relevant models of septic shock (26). The beneficial effect of PPAR-γ activation is likely to be secondary to the inhibition of the production of several inflammatory mediators, as has been shown *in vivo* in septic rodents (26) and *in vitro* in activated macrophages and monocytes (27). Sepsis and other inflammatory states affect the PPAR-γ expression levels and correlate with the inflammatory response. The expression levels of PPAR-γ are downregulated in the lung and vascular endothelium in rodent models of septic shock, and treatment with PPAR-γ ligands reverses the sepsis-induced reduction (26). In adipose tissue, the expression levels of PPAR-γ decreased after the rats were challenged *in vivo* with endotoxins and the cytokine-induced suppression of PPAR-γ was reversed with synthetic agonists (28). However, the mechanisms that lead to a reduction in the levels of PPAR-γ activity in the presence of sepsis remain unclear. In the present study, CLP blocked PPAR-γ expression in the lung tissue, increasing the levels of proinflammatory cytokines; however, the administration of salidroside enhanced the PPAR-γ expression levels in the lung tissue and inhibited the inflammatory response.

To further characterise the inhibitory effect of salidroside on cytokine production, the present study examined the effects of salidroside on the activation of the transcription factor NF-κβ, which regulates the expression of numerous immune and inflammatory genes and the production of cytokines. NF-κβ is essential for host defence and the inflammatory responses to microbial and viral infections (29), as it is an important transcription factor required for the expression of a number of proinflammatory cytokines (30). In the majority of cells, NF-κβ exists in an inactive form in the cytoplasm as it is bound to inhibitory Iκβ proteins. Following CLP challenge, NF-κβ is translocated to the nucleus to drive the expression of a variety of inflammatory genes that are involved in the pathogenesis of ALI. Therefore, a blockage of NF-κβ activation and an increase in the Iκβ expression levels is expected to attenuate ALI (30). This is supported by the results of the present study, which demonstrated that salidroside treatment following the CLP challenge inhibited NF-κβ activation, and the release of inflammatory cytokines promoted the expression of PPAR-γ. This is consistent with the theory that salidroside prevents the release of LPS-induced inflammatory cytokines via its anti-NF-κβ activity, which upregulates PPAR-γ expression levels.

CLP stimulates macrophages, neutrophils and other types of immune cell to produce different mediators, including cytokines such as TNF-α and IL-6, that recruit polymorphonuclear neutrophils to the injured site and contribute to the pathogenesis of ALI and ARDS (31). Activated neutrophils that release various types of mediators and secrete the elastase enzyme, whose activity is an indicator of neutrophil accumulation in tissues (32), are recognised to be a primary mechanism in the development of ALI. In the present study, the interstitial space was shown to be filled with activated alveolar macrophages and neutrophils following the LPS challenge. These pathological changes were reversed by salidroside treatment following the challenge, indicating that salidroside may inhibit LPS-induced leukocyte rolling and transmigration into the lung tissue.

In addition, vascular leakage is a critical pathological process in sepsis (33). Leakage permits plasma protein and leukocyte extravasation, leading to oedema and inflammatory reactions in the affected tissues (34). Oedema causes tissue hypoxia, and leukocytes, including neutrophils, cause tissue damage through their excessive production of free radicals and proteases. Thus, vascular leakage is a promising target for the therapeutic treatment of sepsis. In the present study, CLP was found to markedly increase the albumin concentration in BALF, the W/D lung weight ratio and the water content in lung tissue. Histological analyses of the lungs following CLP exposure revealed a damaged alveolar structure with evident concretions leaking liquid within the bleeding inflammatory cells. In addition, the perivascular gap was widened and there were numerous alveolar stoma infiltrated by mononuclear inflammatory cells. These observations indicated that CLP exacerbates the lung leakage permeability; however, the exacerbated lung leakage permeability was ameliorated by salidroside. These results provide supporting evidence that salidroside post-treatment is effective in reversing LPS-induced lung permeability and injuries.

In conclusion, the observations of the present study clearly demonstrate the anti-inflammatory activity of salidroside via increasing PPAR-γ activation. Furthermore, PPAR-γ was shown to block CLP-induced NF-κβ expression, which consequently upregulated the expression levels of Iκβ. The upregulation of Iκβ expression levels inhibited the accumulation of inflammatory cells within the lungs, improving CLP-induced lung permeability and alleviating the pathological injury of lung tissue in rats with sepsis. Therefore, the present study has provided an experimental basis involving animals for the treatment of sepsis by the administration of salidroside.

## Figures and Tables

**Table I:** Primer sequences of the genes used to validate the microarray analysis by qPCR.

Gene	Primer sequence	Length (bp)
PPAR-γ	F: 5′-ACAAGGACTACCCTTTACTGAAATTACC-3′ R: 5′-GTCTTCATAGTGTGGAGCAGAAATGCTG-3′	178
NF-κβ	F: 5′-GCACGGATGACAGAGGCGTGTATAAGG-3′ R: 5′-GGCGGATGATCTCCTTCTCTCTGTCTG-3′	420
Iκβ	F: 5′-TGCTGAGGCACTTCTGAG-3′ R: 5′-CTGTATCCGGGTGCTTGG-3′	421
β-actin	F: 5′-GATTACTGCTCTGGCTCCTGC-3′ R: 5′-GACTCATCGTACTCCTGCTTGC-3′	190

qPCR, quantitative polymerase chain reaction; PPAR-γ, peroxisome proliferator-activated receptor γ; NF-κβ, nuclear factor-κβ; Iκβ, inhibitor-κβ; F, forward; R, reverse.

## Abbreviations

PPAR-γ: peroxisome proliferator-activated receptor γ
NF-κβ: nuclear factor-κβ
IL: interleukin
TNF-α: tumour necrosis factor-α
LPS: lipopolysaccharide
CLP: cecal ligation and puncture
qPCR: quantitative polymerase chain reaction
ALI: acute lung injury
BALF: bronchoalveolar lavage fluid
ARDS: acute respiratory distress syndrome
ELISA: enzyme-linked immunosobent assay
Iκβ: inhibitor-κβ
RT: room temperature
W/D: wet to dry
ANOVA: analysis of variance
